# Upside-down stomach in paraesophageal hernia: A case report

**DOI:** 10.1097/MD.0000000000036734

**Published:** 2023-12-22

**Authors:** Xiuliang Zhu, Chengyu Hu, Weihua Gong

**Affiliations:** a Department of Radiology, Second Affiliated Hospital of Zhejiang University School of Medicine, Hangzhou, China; b Department of Surgery, Second Affiliated Hospital of School of Medicine, Zhejiang University, Hangzhou, China; c Department of Gastrointestinal Surgery, Second Affiliated Hospital of Zhejiang University School of Medicine, Hangzhou, China.

**Keywords:** hiatal hernia, paraesophageal hernia, upside-down stomach

## Abstract

**Rationale:**

Paraesophageal hernias, accounting for a mere 5% to 10% of all hiatal hernias, occasionally present an exceedingly uncommon yet gravely consequential complication characterized by the inversion of the stomach. Delving into the clinical manifestations and optimal therapeutic approaches for patients afflicted by this condition merits substantial exploration.

**Patient concerns:**

A 60-year-old man was referred to our hospital with acute onset of severe epigastric pain, abdominal distension, and vomiting. A chest radiograph unveiled an elevated left diaphragmatic dome accompanied by a pronounced rightward shift of the mediastinum. Subsequent abdominal computed tomography imaging delineated the migration of the stomach, spleen, and colon into the left hemithorax, facilitated by a significant diaphragmatic defect.

**Diagnoses:**

The diagnosis of a giant paraesophageal hernia with complete gastric inversion was established through a comprehensive evaluation of the patient’s clinical manifestations and imaging findings.

**Interventions:**

Surgical intervention was performed on the patient. During the procedure, a left diaphragmatic defect measuring approximately 10 × 8 cm was identified and meticulously repositioned, followed by the repair of the diaphragmatic hernia. The herniated contents comprised the pancreas, stomach, spleen, a segment of the colon, and a portion of the greater omentum.

**Outcomes:**

The patient experienced a smooth postoperative recuperation and was discharged 12 days following the surgical procedure. Subsequently, during a 7-month follow-up period, the patient continued to exhibit favorable progress and recovery.

**Lessons:**

Paraesophageal hernias are rare, and the presence of an inverted stomach in a giant paraesophageal hernia is exceptionally uncommon. Clinical presentation lacks distinct features and can lead to misdiagnosis. This case emphasizes the importance of timely surgical intervention guided by imaging, offering valuable clinical insights.

## 1. Introduction

Upside-down stomach anomaly is an infrequent manifestation of paraesophageal hernia, constituting a minority subset within the realm of diaphragmatic hernias. Among the spectrum of hiatal hernias, paraesophageal herniation comprises a mere 5% to 10%. The presence of an upside-down stomach is exceptionally rare, and the prompt identification of this anomaly in its incipient stages holds substantial clinical significance, contributing significantly to the formulation of effective treatment strategies. When a large paraesophageal hernia occurs, the abdominal organs protrude into the thoracic cavity through the hiatal hernia. The condition known as upside down stomach is considered a severe manifestation of gastric volvulus, which was first documented by Berti in 1886. Gastric volvulus can occur along the organ and mesenteric axes, with the latter being relatively uncommon.^[1,2]^ In the acute phase, upside down stomach primarily presents with gastrointestinal symptoms. It is crucial to promptly and accurately diagnose this condition, as ischemia, necrosis, and perforation may be observed in cases of incarcerated stomach with gastric volvulus.^[3]^ Timely surgical intervention significantly mitigates mortality and associated complications. This case report contributes substantial clinical evidence to enhance treatment and diagnostic approaches.

## 2. Case presentation

**Figure 1.**
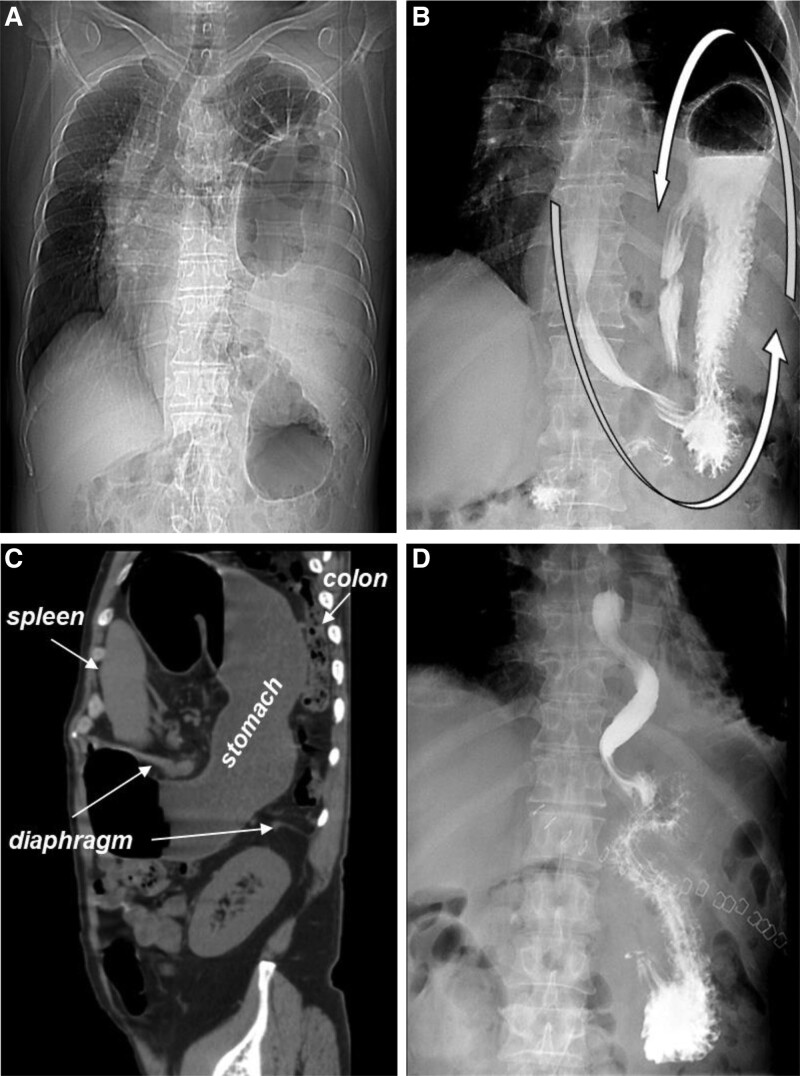
(A) Chest radiograph shows elevated left diaphragmatic dome with rightward shift of mediastinum. (B) Upper gastrointestinal barium study shows stomach was upside-down. (C) Abdominal CT reconstructed sagittal image shows stomach, spleen, and colon passing through left hemithorax via large diaphragmatic defect. (D) Postoperative barium meal imaging indicates stomach’s return to normal position.

A 60-year-old man was referred to hospital due to an acute episode of severe epigastric pain, abdominal distension, and vomiting that lasted tens of hours. This patient has no previous gastrointestinal or cardiovascular disease, and no history of abdominal and thoracic trauma was evident in anamnesis. While patient has a history of high blood pressure and takes medication regularly. The patient exhibited mild tenderness of the epigastrium and left upper quadrant with guarding and no rebound tenderness on physical examination. Cardiovascular and respiratory examinations were unremarkable. The routine laboratory studies were unremarkable. Hence, chest radiography (Fig. 1A), upper gastrointestinal barium study (Fig. 1B), and abdominal computed tomography (CT) scanning (Fig. 1C) was performed. Chest radiograph showed a high left diaphragmatic dome and the mediastinum has shifted to the right. Upper gastrointestinal barium study showed that the stomach was completely upside-down. Abdominal CT reconstructed sagittal image revealed large diaphragmatic defect which allowed passage of the stomach, colon, and spleen in the left hemithorax. Based on the patient’s clinical symptoms and imaging manifestations, the diagnosis of diaphragmatic hernia was made. Emergency laparotomy was performed and a left diaphragmatic defect measuring about 10 × 8 cm was identified. Hernia contents involving the pancreas, stomach, spleen, part of the colon, and the greater omentum were reduced. A splenectomy and gastropexy were performed and primary repair of diaphragm with mesh was done. Postoperative upper gastrointestinal barium study was normal (Fig. 1D). The postoperative course was uneventful, and the patient was discharged 12 days after surgery and was well at 7 months’ follow-up. Although the incidence is low, when it occurs there is a high risk of life-threatening complications. By describing the clinical profile and treatment options for this rare disease, it provides direction and experience for clinicians in diagnosis and treatment. This case will also enhance our knowledge and understanding of the disease and avoid misdiagnosis.

## 3. Discussion

Diaphragmatic hernia is categorized into congenital and acquired. Among the acquired types, esophageal hiatal hernia is prevalent. There exist 4 categories of esophageal hiatal hernias: sliding, paraesophageal, mixed, and giant paraesophageal.^[4]^ However, a standardized definition for giant paraesophageal hernias is lacking. Some assert that when at least 30% of the stomach translocates into the thoracic cavity, it qualifies, while others posit a threshold of over 50% stomach hernia.^[5]^ In this presented case, the paraesophageal hernia displayed as an upside-down stomach, or even herniation of the spleen, colon, small intestine, or pancreas into the thoracic cavity. This signifies a comprehensive stomach prolapse, warranting the diagnosis of a type IV giant paraesophageal hernia. Upside-down stomach occurrence within paraesophageal hernias is exceptionally rare, prompting discussion on potential pathogenesis and clinical manifestations.

Diaphragmatic hiatus and relaxation of the phrenoesophageal membrane stand as primary culprits for diaphragmatic herniation.^[6]^ The presence of a pressure gradient between thoracic and abdominal cavities facilitates herniation of the stomach and other abdominal organs into the thoracic region. The stomach and other organs rotate into the thoracic cavity along the axis of the diaphragmatic membrane organ from the esophageal hiatus. As this progression unfolds, the stomach undergoes a 180-degree rotation, culminating in gastric inversion. Research has demonstrated that with age, the diaphragmatic esophageal ligament’s resilience and tension decline, primarily attributed to decreased collagen levels.^[7]^ Consequently, the incidence of esophageal hiatal hernia significantly rises in elder patients harboring concurrent ailments and degenerative conditions. Further investigations have suggested the potential role of genetic factors as etiological contributors.^[8,9]^ In a clinical context, symptoms associated with gastroesophageal reflux disease might manifest in type I and type II hiatal hernias. Type III hernias usually present with epigastric pain, nausea, and dysphagia. Notably, type IV giant paraesophageal hernias can induce dyspnea, abdominal pain, bloating, and vomiting, although certain patients remain asymptomatic.^[10]^ The clinical portrayal of an upside-down stomach hinges upon disease progression, stomach location, gastric torsion, and the extent of obstruction. The acute phase is typified by gastrointestinal obstruction, which underlines the urgency of diagnosis and intervention.^[11]^ With the stomach twisted and inverted within the thoracic cavity, bleeding of the gastric mucosa ensues, representing a significant pathological consequence. The gravest complications involve gastric perforation or necrosis. Furthermore, gastric herniation has been linked to the onset of iron deficiency anemia. This condition arises due to chronic blood loss attributed to mucosal lesions stemming from the stomach’s twisting process, an aspect often underestimated, ultimately culminating in iron deficiency anemia.^[12]^ Hernia sacs that protrude into the chest cavity can result in the constriction of the thoracic dimensions. This occurrence compromises both the respiratory and circulatory systems due to the resultant compression exerted on the heart and lungs.^[13,14]^

Chest X-ray, CT, and upper gastrointestinal barium studies serve as diagnostic aids to corroborate the diagnosis. Among these, CT is hailed as the diagnostic gold standard for hernias.^[15]^ It affords clinicians the precision to delineate hernia location, dimensions, and contents. Recent series of cases have reported a mortality rate of approximately 15% to 20% for paraesophageal hernias, with a noteworthy surge in mortality when severe complications arise.^[16]^ This underscores the criticality of emergent surgical hernia repair as the chosen course of action. The transition from traditional open surgery to laparoscopic treatment represents a substantial advancement, bearing significant implications for hiatal hernia treatment.^[17]^ Presently, laparoscopic fundoplication stands as a prevalent strategy for addressing paraesophageal hernias. The utilization of laparoscopic techniques boasts favorable attributes including a high success rate, expedited recovery, and fewer postoperative complications.^[18–20]^ Previously, nonabsorbable suture repair was the norm for most paraesophageal hernia surgical interventions. Despite ongoing debates concerning complications arising from mesh repair, biological meshes have garnered increased attention due to their merits, including reduced recurrence rates and heightened resistance to infection.^[21]^

In contrast to earlier case reports, this patient’s clinical presentation centered solely around severe epigastric pain, abdominal distension, and vomiting. Interestingly, the absence of prominent symptoms like cardiac compression and dyspnea indicates that during the acute phase of a giant paraesophageal hernia, gastrointestinal symptoms could assume a primary role. This underscores the immediate need to accurately confirm acute giant paraesophageal hernia cases, averting any potential misdiagnoses that might compromise effective treatment strategies. Age-related factors should be considered, as diminished diaphragmatic tone and ligament laxity likely contributed to the abrupt onset of acute paraesophageal hernia. A noteworthy observation is that the patient exhibited a complete inversion of the stomach within the thoracic cavity, accompanied by concurrent herniation involving the pancreas, spleen, colon, and greater omentum. Fortunately, swift intervention through laparoscopic repair surgery culminated in a positive prognosis for the patient. Distinguishing this case from previous reports is the exceptional rarity of the patient’s giant paraesophageal hernia, positioning it as a valuable model for shaping future diagnostic and therapeutic frameworks.

Given the infrequent occurrence and increased risk associated with upside-down stomach in paraesophageal hernias, the main focus is on swift and timely diagnosis, coupled with surgical intervention. This approach significantly reduces patient mortality rates and improves overall prognoses. The underlying mechanisms causing inverted stomach in paraesophageal hernias need thorough exploration in subsequent research efforts. This can lead to proactive strategies to prevent upside-down stomach from emerging in this clinical context. Furthermore, establishing standardized protocols for clinical diagnosis and treatment is crucial. This ensures consistency and accuracy in patient care. The ongoing exploration of various treatment methods not only represents optimism but also instills renewed hope among those affected by this condition.

## Author contributions

**Conceptualization:** Xiuliang Zhu.

**Supervision:** Weihua Gong.

**Writing – original draft:** Xiuliang Zhu.

**Writing – review & editing:** Chengyu Hu.
